# Texas State Medical Association; a Card to Members of Local Societies

**Published:** 1896-07

**Authors:** 


### Texas State Medical Association.

One of the most sensible things done by the State Association at the recent meeting at Fort Worth, was the adoption of a plan whereby all members of local societies are brought into affiliation with the State Association, membership in the home society virtually making them members of the State Association, merely upon the payment of the yearly dues. This enables one to become an active member of the State Association without being compelled to be present, or to even submit diploma (which can be done only once a year) at the annual meeting. In other words, there is but one gate of admittance to membership.

Upon making application for membership in his home society, it is presumed that one's credentials are carefully examined, and that due regard has been had to the character and standing of the applicant; that the censors of the local society are always competent to pass on qualifications for membership, and that they observe proper care in so doing; and in that way it is concluded that no unworthy person is admitted. Upon this assumption, a certificate of membership in any county or district society will admit a person to full fellowship in the State Association at any time. It is practically the same plan that is in operation with the American Medical Association—membership in a State organization entitling a person to membership in the National Association upon the payment of the dues. The zealous Secretary, Prof. H. A. West, of the Medical Department, University of Texas, Galveston, is sending out slips, of which we give a copy below.

This is a "step in the right direction," and calculated, we hope and believe, to add greatly to the membership and usefulness of the Association. There is one precaution to be observed, however. A circular should be issued to local societies enjoining them to be strict in the observance of the necessary precautions before admitting an applicant. In a great majority of cases a diploma is the only requisite, and unworthy men are often admitted. Once in, it is difficult to get them out, as the State Association realizes by now. Not only unworthy men, but illiterate men are admitted. Men almost wholly without literary education may, and often do, obtain medical education more or less—and are granted a diploma. The writers of those letters published elsewhere in this issue, are graduates of reputable medical schools. It is "a mystery how they do it, yet they do;" how the colleges granting a diploma to such men, get over or around the requirement, "evidences of a good English education."

Office of the Secretary,
Galveston, Texas.

Dear Doctor:—In the interest of a united profession you are earnestly solicited to become a member of the State Medical Association. The initiation fee has been dispensed with, and you may now secure membership without attendance upon the meetings, by the payment of $5 and returning this slip to the Treasurer. You will receive the transactions if enrolled by the first of July.

Fraterally,

H. A. West, Secretary.

